# De Novo Hepatic Transcriptome Assembly and Systems Level Analysis of Three Species of Dietary Fish, *Sardinops sagax*, *Scomber japonicus*, and *Pleuronichthys verticalis*

**DOI:** 10.3390/genes9110521

**Published:** 2018-10-25

**Authors:** Dylan J. Richards, Ludivine Renaud, Nisha Agarwal, E. Starr Hazard, John Hyde, Gary Hardiman

**Affiliations:** 1 Bioengineering Department, Clemson University, Charleston, SC 29425, USA; djricha@clemson.edu; 2 Department of Medicine, Medical University of South Carolina, Charleston, SC 29425, USA; renaudl@musc.edu; 3 Center for Genomic Medicine, Bioinformatics, Medical University of South Carolina, Charleston, SC 29425, USA; hazards@musc.edu; 4 Biomedical Informatics Research Center, San Diego State University, San Diego, CA 92182, USA; nisha2683@gmail.com; 5 Academic Affairs Faculty & Computational Biology Resource Center, Medical University of South Carolina, Charleston, SC 29425, USA; 6 NOAA Fisheries, Southwest Fisheries Science Center, La Jolla, CA 92037, USA; john.hyde@noaa.gov; 7 Department of Public Health Sciences, Medical University of South Carolina, Charleston, SC 29425, USA; 8 Laboratory for Marine Systems Biology, Hollings Marine Laboratory, Charleston, SC 29412, USA; 9 School of Biological Sciences & Institute for Global Food Security, Queens University Belfast, Stranmillis Road, Belfast BT9 5AG, UK

**Keywords:** sardine, mackerel, turbot, RNA sequencing, de novo transcriptome assembly, genomic biomarkers

## Abstract

The monitoring of marine species as sentinels for ecosystem health has long been a valuable tool worldwide, providing insight into how both anthropogenic pollution and naturally occurring phenomena (i.e., harmful algal blooms) may lead to human and animal dietary concerns. The marine environments contain many contaminants of anthropogenic origin that have sufficient similarities to steroid and thyroid hormones, to potentially disrupt normal endocrine physiology in humans, fish, and other animals. An appropriate understanding of the effects of these endocrine disrupting chemicals (EDCs) on forage fish (e.g., sardine, anchovy, mackerel) can lead to significant insight into how these contaminants may affect local ecosystems in addition to their potential impacts on human health. With advancements in molecular tools (e.g., high-throughput sequencing, HTS), a genomics approach offers a robust toolkit to discover putative genetic biomarkers in fish exposed to these chemicals. However, the lack of available sequence information for non-model species has limited the development of these genomic toolkits. Using HTS and de novo assembly technology, the present study aimed to establish, for the first time for *Sardinops sagax* (Pacific sardine), *Scomber japonicas* (Pacific chub mackerel) and *Pleuronichthys verticalis* (hornyhead turbot), a de novo global transcriptome database of the liver, the primary organ involved in detoxification. The assembled transcriptomes provide a foundation for further downstream validation, comparative genomic analysis and biomarker development for future applications in ecotoxicogenomic studies, as well as environmental evaluation (e.g., climate change) and public health safety (e.g., dietary screening).

## 1. Introduction

The monitoring of marine species as sentinels for ecosystem health has long been a valuable tool worldwide, giving insight into how both anthropogenic pollution and naturally occurring phenomena (i.e., harmful algal blooms) may lead to human and animal dietary concerns [1,2,3,4,5,6,7,8]. In addition to global influences of human activity on the environment (e.g., climate change), the coastal and estuarine environments contain many organic and inorganic contaminants from anthropogenic effluents [9]. Many of these contaminants have sufficient structural and chemical similarities to steroid and thyroid hormones, suggesting that they are potentially able to disrupt normal endocrine physiology in humans, fish, and other animals. An appropriate understanding of the effects of these endocrine disrupting chemicals (EDCs) on forage fish (e.g., sardine, anchovy, mackerel) can lead to significant insight into how these contaminants may affect local ecosystems in addition to their potential impacts on human health.

The San Diego South Bay region, which includes the cities of Coronado, Imperial Beach, and Tijuana, Mexico, has many diverse sources of pollution to the marine environment of both U.S. and Mexican origin leading to a long history of serious and ongoing water quality issues that have significant human health impacts [10,11,12,13]. This region is exposed to chemicals from domestic, agricultural, and industrial sources [14] which enter the coastal environment though the South Bay Ocean Outfall (SBOO), the Tijuana River Estuary, and other point and non-point sources. As the production of these compounds is likely to continue, there is increasing concern about the short- and long-term impacts of EDC exposure on human populations [15,16,17].

In this study, we focused on three common species of fish present in the marine environment of the San Diego South Bay region. Pacific sardine (*Sardinops sagax*) is a schooling coastal pelagic fish, often comprising the bulk of the diet for predatory vertebrates in this coastal ecosystem including several federally protected species (e.g., California least tern, brown pelican, bottlenose dolphin) [18]. This species is present along the west coast of North America, existing across gradients of temperature, salinity, and anthropogenic pollution [19]. As filter feeders, these coastal plankton feeders can readily accumulate toxins from the environment [19]. The Pacific chub mackerel (*Scomber japonicus*) overlaps in habitat with Pacific sardine, but feeds at a slightly higher trophic level on copepods, crustaceans, juvenile fishes, and squid. This species has been shown to be an effective sentinel species that helped to evaluate the effects of the Deepwater Horizon oil spill, which released crude oil and contaminated surface water habitats for pelagic fish for more than 12 weeks [20]. The hornyhead turbot (*Pleuronichthys verticalis*) is a demersal flatfish that resides on the mainland shelf from southern Baja California, Mexico to northern California, feeding primarily on sedentary tube-dwelling polychaetes that are present in sediment [21,22]. As sediment-associated and benthic feeders, hornyhead turbot are good sentinels for pollution monitoring due to their higher risk of exposure to chemicals that accumulate in sediments. Moreover, they inhabit a limited area, which allows one to localize the point source of chemical pollution [23]. In addition to their utility as sentinels for ecosystem health, these fish are also popular and important food sources for humans, owing to their rich omega-3 fatty acid content and flavor [24].

As a means to link bioavailability measurements for EDCs and fish response, a genomics approach offers a robust toolkit to associate the presence of these chemicals with genomic biomarkers. Advances in molecular methods, such as microarrays, quantitative PCR (q-PCR) and high-throughput sequencing (HTS) have substantially improved the sensitivity of analyses for assessing the biological effects of EDCs on animal physiology [23,25,26,27,28,29,30]. Recently developed genomics technologies have also facilitated assessment of the effects of xenobiotics in the environment on human health [31]. However, the paucity of available sequence information for non-model species is a challenge in developing these genomic toolkits.

The present study aimed to establish, for the first time for these three species, a de novo global transcriptome database for the liver, the primary organ involved in detoxification using HTS and de novo assembly technology. By combining the RNA-Seq output of liver samples from each of the three species of fish, we assembled and annotated their hepatic transcriptomes. The assembled transcriptomes provide a foundation for further downstream validation, comparative genomic analysis and biomarker development for future applications in ecotoxicogenomic studies, as well as environmental evaluation (e.g., climate change) and public health safety (e.g., dietary screening).

## 2. Materials and Methods

### 2.1. Fish Sampling

Hornyhead turbot, sardine, and mackerel were chosen for study as they are common in the San Diego region and are species that have been used in California seafood contaminant monitoring programs. Fish were obtained either from a local commercial live bait provider (Everingham Brothers, La Mesa, CA, USA) or by bottom trawl (hornyhead turbot), all fish originated from the San Diego region. Fish (hornyhead turbot) collections were approved by and conducted under scientific collection permits issued by the California Department of Fish and Wildlife. This species is not threatened or endangered, and the collection sites were not located in ecological reserves or areas receiving special ecological protection. The sampling method (otter trawl) limits bycatch mortality by using a relatively small net, holding the catch in flowing seawater, and promptly returning non-target individuals to the ocean. These methods are the same as those used in regional monitoring programs, which have been approved by local and federal fish and wildlife and regulatory agencies [27,32]. Humane handling of the fish was assured by the use of a Standard Operating Procedure developed specifically for the study and approved by a Steering Committee composed of the study participants. Fish were transferred rapidly to holding tanks with flowing and aerated seawater at ambient temperature (~17 °C) for a minimum of 30 days prior to experiments to ensure that the fish show no signs of disease or injury and had recovered from initial handling and transport stress. Fish were humanely euthanized using either an overdose of tricaine methanosulfonate (250 mg/L) dissolved in seawater or by cervical dislocation prior to dissection. The livers were harvested and frozen in liquid nitrogen and stored at −80 °C.

### 2.2. RNA Extraction

Isolation of total RNA from liver samples was performed using TRIzol reagent (Invitrogen/Thermo Fisher Scientific, Waltham, MA, USA) and the extracted RNA were further purified using the RNeasy Mini kit (Qiagen, Valencia, CA, USA). All RNA samples were subjected to on-column digestion of DNA during RNA purification from cells, to ensure highly pure RNA-free from DNA contamination. The concentrations were determined by absorbance readings (OD) at 260 nm using an ND-1000 (Nanodrop, Wilmington, DE, USA). RNA was further assessed for integrity with the 6000 Nano LabChip assay from Agilent (Santa Clara, CA, USA). Only RNA samples with a RNA Integrity Number (i.e., RIN) score of >7.0 were used for RNA-Seq.

### 2.3. High-Throughput Sequencing

For the RNA-Seq experiments, five individual liver samples from each species were pooled. To prepare mRNA-Seq libraries, the TruSeq RNA Sample Prep Kit (Illumina, San Diego, CA, USA) was utilized; 100–200 ng of total input RNA was used in accordance with the manufacturer’s protocol. High-throughput sequencing was performed using Illumina instruments (GAIIX or HiSeq2000) with each species’ mRNA library sequenced to a minimum depth of ~8 million reads. A 100 bp single-end sequencing strategy was employed. Data were subjected to Illumina quality control procedures (>80% of the data yielded a Phred score of 30).

All sequencing data has been uploaded to the Sequence Read Archive (SRA) database under reference PRJNA493102.

### 2.4. De Novo Assembly and Transcriptome Annotation

**Figure 1:**
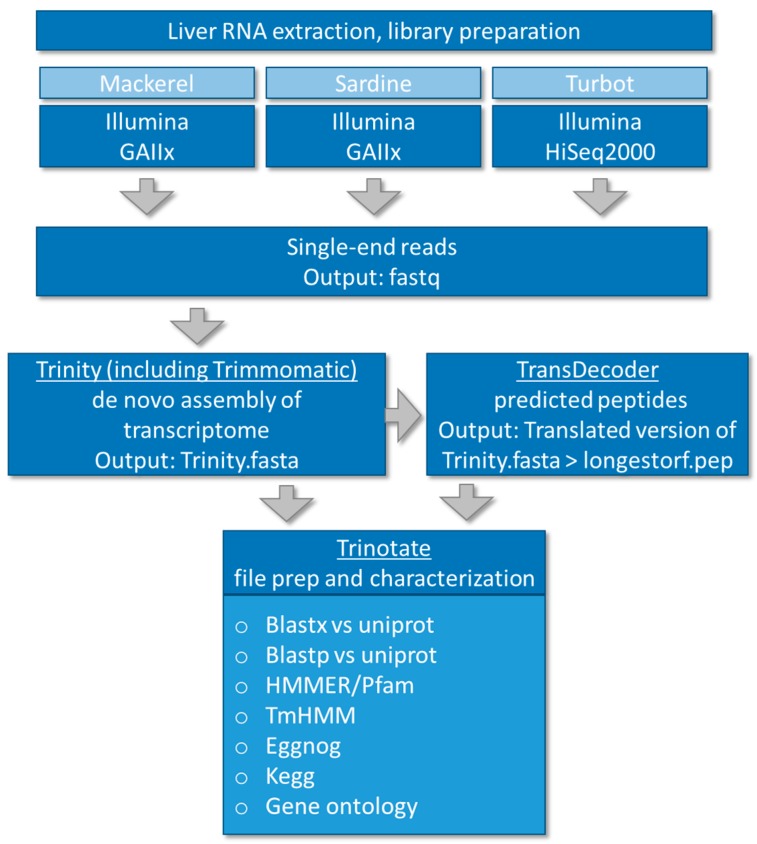
Workflow of de novo transcriptome assembly and annotation of three dietary fish species, mackerel (*Scomber japonicas*), sardine (*Sardinops sagax*), and turbot (*Pleuronichthys verticalis*).

Illumina adapter sequences and sequences that did not meet quality thresholds were removed using Trimmomatic [33]. All reads were pooled and assembled de novo using Trinity (version 2.2.0) with *k*-mer length set at 25 according to the original default strategy [34]. Trans-decoder (version 3.0.0) was used for coding sequence prediction [35], and Trinotate (2.0.2) for functional annotation [36]. Trinotate is a comprehensive annotation suite specifically designed for automatic functional annotation of de novo assembled transcriptomes of non-model organisms, including homology search to known sequence data (BLAST+/SwissProt) [37], protein domain identification (HMMER/PFAM) [38], transmembrane domain prediction (tmHMM) [39,40], and leveraging various annotation databases (eggNOG/GO/KEGG databases) [41,42,43,44,45], reporting the best hits in the databases (http://trinotate.github.io/). The eggNOG database is based on the original idea of COGs (clusters of orthologous groups), the KEGG (Kyoto Encyclopedia of Genes and Genomes) database is a collection of databases with genomes, biological pathways, diseases, drugs, and chemical substances and the Gene Ontology (GO) project provides functional information in the context of cellular component, (i.e., the parts of a cell or its extracellular environment), molecular function, (the activities of a gene product at the molecular level, e.g., binding or catalysis, and biological process (molecular events with a defined beginning and end). A summary data file of each fish annotation file (i.e., non-redundant Trinotate output) can be found in Supplementary Tables S1–S3. The Trinity fasta assemblies were analyzed for annotation and completeness with the BUSCO [46,47] program with the Actinopterygii_odb9 [48] dataset with zebrafish as reference. Bundled software with the BUSCO2 virtual machine included a UBUNTU image, NCBI BLAST, Augustus software version 3.2.2. [49] (http://bioinf.uni-greifswald.de/augustus/references), and hmmer version hmmer-3.1b2-linux-intel-ia32 (http://www.hmmer.org). BUSCO2 was run as a virtual machine via the Oracle VirtualBox software (https://www.virtualbox.org/). All analysis was carried out on a local high-performance compute cluster at the Medical University of South Carolina. The overall workflow is summarized graphically in Figure 1.

The annotation depth of zebrafish (*Danio rerio*) and humans (*Homo sapiens*) was collected from the Gene Ontology Consortium over four time points and the ratio of human to zebrafish was taken for each metric [45,50].

### 2.5. Systems Level Analysis

Blastp hits were compared for overlap using area-proportional Venn diagrams created using VENNY 2.1 [51]. To gain a functionalized understanding of the assembled transcriptomes, gene ontology (GO) for biological process were obtained from the list of blastp hits from each fish. After gathering a list of blastp hits per fish, species origin identifiers were removed (e.g., GAPDH_HUMAN -> GAPDH), and all duplicates were removed, so as to reduce overlapping redundancies in the functional analysis. When using zebrafish as the background annotation, the GO online tool g:Profiler was used with default settings with *p* < 0.05 and statistical domain size of “all known genes” [52]. Human annotated GO queries were performed using ToppFun from the ToppGene Suite with default settings [53]. GO terms with *p* < 0.05 were then visualized in semantic similarity-based scatterplots using REduce & VIsualize Gene Ontology (REViGO) that combines redundant terms into a single, representative term based on a simple clustering algorithm relying on semantic similarity measures [54]. Selected GO terms were highlighted to indicate overall similarities and spatial organization.

## 3. Results

### 3.1. De Novo Assembly of Transcriptomes Using Trinity

**Figure 2:**
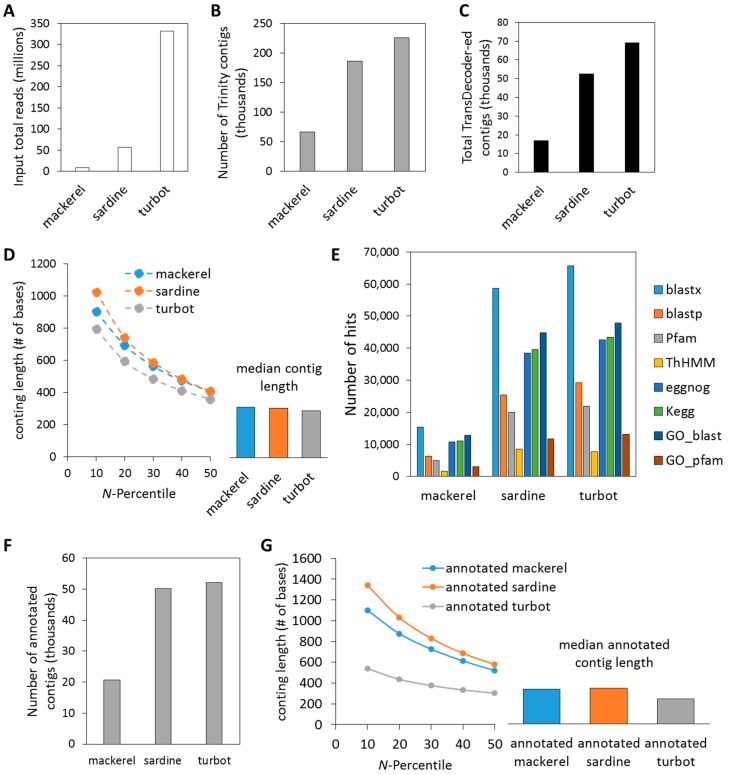
Quantitative Trinotate summary of Trinity transcriptome assembly output. (**A**) Each fish had unique number of input total number of reads that resulted in (**B**) differing number of resulting total assembled Trinity contigs and (**C**) total TransDecoder-ed contigs. (**D**) *N*-percentiles and median length of total contigs. (**E**) Sardine and turbot showed notable increases in non-redundant annotation hits over mackerel, attributed to differences in sequencing coverage. (**F**) Number of contigs with at least one annotation hit. (**G**) Re-evaluation of *N*-percentiles and median length of contigs with at least one annotation hit.

By combining the RNA-Seq output of liver samples from each of the three fish species, we were able to assemble and annotate novel transcriptomes (Figure 1). As seen in Figure 2 and summarized in Supplementary Table S4, sequencing coverage (i.e., total input reads) was highest for turbot (T: 331,569,698), followed by sardine (S: 56,432,715) and mackerel (M: 8,868,248) (Figure 2A), which resulted in differing numbers of total assembled contigs and total TransDecoder contigs/peptides (Figure 2B,C). This also reflected the sequencing instrument utilized and the depth to which each sequencing library was sequenced. Despite differences in sequencing coverage, de novo assembly using Trinity produced similar sized contigs in each *N*-percentile category with the median contig length ~300–400 bp for each of the three species (Figure 2D). Increased sequencing coverage did, however, provide increased amounts of GO, evolutionary genealogy of genes and pathway annotated information, as seen in the Trinotate annotation hits output (Figure 2E, Supplementary Tables S1–S3). Based on the final annotation outcome, assembled contigs were re-characterized after selecting for Trinity contigs with at least one annotation hit derived from the Trinotate characterization for each fish (i.e., annotated contigs) (Figure 2F). This resulted in a higher *N*-percentile and median numbers for mackerel and sardine, while slightly decreasing numbers for turbot (Figure 2G). In support of increased annotation terms with increased sequencing coverage, Benchmarking Universal Single-Copy Orthologs (BUSCO) analysis for the completeness of assembled transcriptomes showed that the lower sequence coverage (i.e., mackerel) had 18% partial/complete BUSCOs, whereas higher sequencing coverage (i.e., sardine, turbot) had ~30% partial/complete BUSCOs (Supplementary Table S5).

### 3.2. Functional Analysis of De Novo Hepatic Transcriptomes

**Figure 3:**
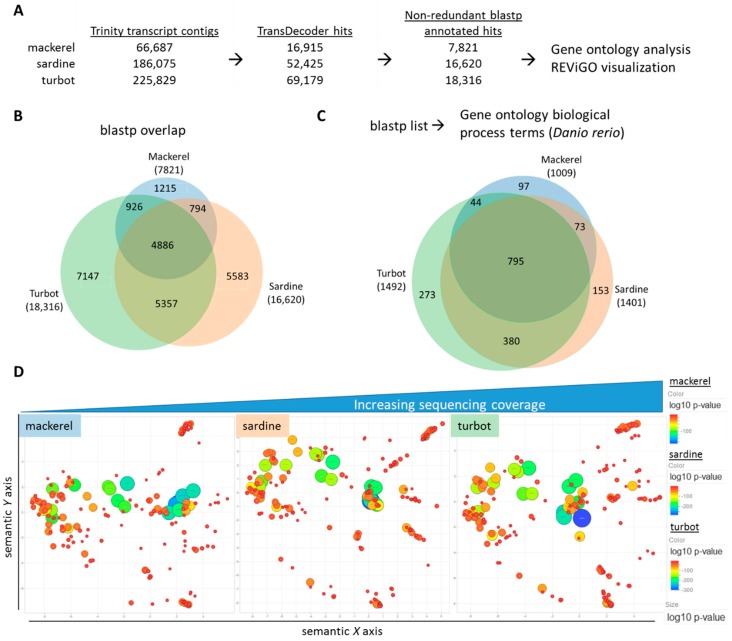
Comparative functional analysis of assembled transcriptomes. (**A**) Workflow of selecting non-redundant blastp hits from original Trinity contigs for comparative functional analysis. (**B**) A Venn diagram showing the overlap of non-redundant, identified hits from blastp query with each total number reflecting sequencing coverage. (**C**) A Venn diagram showing the overlap of gene ontology (GO) terms for biological processes derived from the blastp list for each fish using the zebrafish (*Danio rerio*) species as the annotation background. (**D**) REViGO plot of the top 350 significant GO terms after grouping of redundant terms (colored/sized by *p*-value of GO term category) reveals similar functional spatial distribution of top functional GO terms between fish.

The assembled transcriptomes provided a foundation for further downstream validation and comparative genomic analysis. As each of these samples is derived from the liver of several fish per species, the assembled transcriptomes represented a pooled transcriptomic view of each species and permitted a systems-level analysis on a per species basis. To focus on a functional view of each species, the protein-based annotation (i.e., TransDecoder/blastp) was used for continued analysis using, and a visual workflow can be seen in Figure 3A. The list of identified hits from the individual blastp files were used to assess hepatic gene expression profiles for each of the three fish species and facilitate cross-species comparisons. In Figure 3B, comparison of the blastp hits from each species using a Venn diagram revealed a common set of 4886 genes accounting for ~19% of all genes across all three species (Supplementary Table S6). When using a GO term annotation library based on zebrafish, GO analysis showed an overlap of 795 GO terms representing ~20% of all GO terms (Figure 3C, Supplementary Table S7). As many of the GO terms are likely functionally redundant or closely related, REViGO analysis was used to reduce GO term redundancy, prioritize by GO term *p*-value, and visualize semantic similarity-based scatterplots for each species. By using the top-ranked GO terms by *p*-value (max. 350), REViGO plots revealed a strikingly similar spatial distribution for each species despite the differences in sequencing coverage (Figure 3D, Supplementary Table S8).

**Figure 4:**
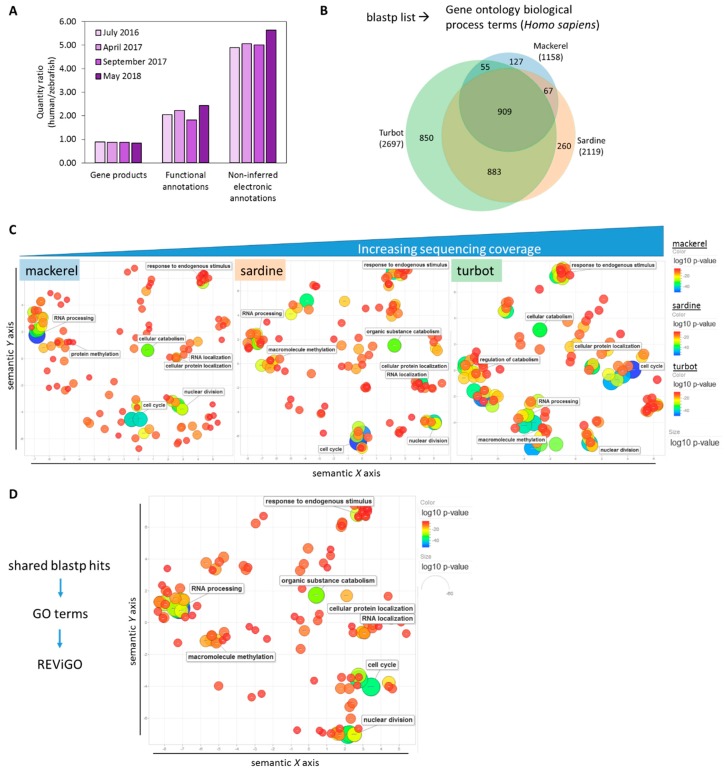
Comparative functional analysis of assembled transcriptomes using *Homo sapiens* annotation background. (**A**) Although gene product quantity is similar, human (*Homo sapiens*) annotation depth continues to be notably higher than zebrafish (*Danio rerio*) annotation based on the ratio of annotation metrics of human/zebrafish (as of May 2018). (**B**) A Venn diagram showing the overlap of gene ontology (GO) terms for biological processes derived from the blastp list for each fish using human (*Homo sapiens*) as the annotation background. (**C**) REViGO plot of the top 350 significant GO terms after grouping of redundant terms reveals similar functional spatial distribution of top functional GO terms between fish despite differences in sequencing coverage. Color (blue) and size (large) indicate low *p*-value of GO term category. (**D**) GO term identification and REViGO plot of overlapping blastp hits produce similar spatial distribution as shown in (**C**), supporting the overall functional similarity across sequencing coverage.

**Figure 5:**
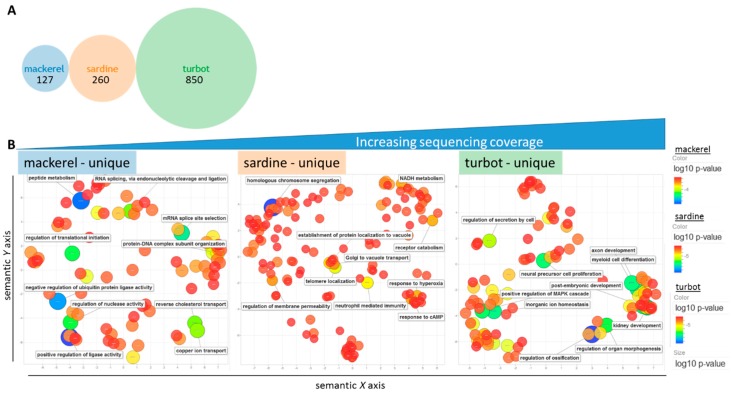
Unique GO terms for each fish. (**A**) Venn diagram showing the number of unique humanized GO terms from previous comparative analysis. (**B**) REViGO plot of the 127, 260, and top 350 significant unique GO terms from mackerel, sardine, and turbot, respectively, after grouping of redundant terms reveals extended information on general physiological terms despite differences in sequencing coverage. Color (blue) and size (large) indicate low *p*-value of GO term category.

In comparison to the depth of available annotation terms for zebrafish, the human (*Homo sapiens*) annotation background is notably larger than previously discussed (Figure 4A) [55]. When the fish genes were projected onto their human orthologs with a human GO term annotation library during GO term analysis, the fish GO terms overlapped by 909 terms (~28% of the total number) and the number of unshared terms was increased (Figure 4B, Supplementary Table S9). With a list based on “humanized” GO terms, REViGO plots of the top-ranked GO terms by *p*-value (max. 350) revealed unique patterning for each fish albeit with a similar overall distribution of related terms, as seen by the representative labeled terms (Figure 4C, Supplementary Table S10). Furthermore, a REViGO plot of the GO terms of the 4886 shared blastp hits from Figure 3A and Supplementary Table S4 showed a similar spatial distribution (Figure 4D), supporting the overall predicted functional similarity between fish liver samples across a range of sequencing coverage. Analysis of unique humanized GO terms showed an increasing number of unique terms according to sequencing coverage (Figure 5A). REViGO plots labeled with the top significant terms showed general physiological function-related terms that may reflect the added insight from increased sequencing coverage (Figure 5B, Supplementary Table S11).

## 4. Discussion

The assembly of these global transcriptomes for three sentinel coastal fish species provides a platform and template for future sequencing-based comparative studies. De novo assembly, as opposed to reference-genome based assembly, offers a direct evaluation of genetic information without interspecies discrepancies or biases. High-throughput screening creates large amounts of short-read data that poses a number of computational challenges in re-assembling. The development of the de Bruijn strategy (central to the Trinity method) to reconstruct transcriptional contigs offered a more accelerated, efficient computational path for next-generation sequencing to assemble large amounts of short-read data [56]. Given the current use of short-reads for sequencing, Trinity has been shown to be a robust and accurate method to construct de novo assemblies no matter the sequencing coverage [57]. Nevertheless, as sequencing strategies are further developed, the optimized method of assembly should take into account the sequencing parameters (e.g., depth, read length, genome size) and the overall purpose of the study (e.g., toxicity screening, splice variant exploration) [58].

In the context of toxicological studies, as systems-level/global molecular tools (e.g., RNA-Seq) become more available, genomic characterization will serve to provide an important view of the effects of contaminants, such as EDCs, and environmental shifts on fish health [59,60,61,62]. Here, by focusing on the shared genes and biological processes in the hepatic transcriptomes of three different fish species, we showed that major physiological functions can be observed and tracked at the genetic level despite differences in sequencing coverage and fish habitat. This contributes to the growing understanding of how sequencing coverage influences the downstream analysis. Notably, lower coverage, such as in the mackerel samples, could allow for high-throughput analysis in the context of transcriptomic screening, while retaining the ability to track changes in major biological processes. It is important to note that while overlapping genes may serve as the foundation to indicate relevant functions across species, trophic levels, and/or habitats, increased coverage provided extended details that may be useful in a targeted study, especially if the target environmental contaminants-affected transcript is not represented in low coverage data. Furthermore, additional optimization techniques of de novo assembly of non-model organisms may enhance transcriptomic information, supporting downstream analysis [63,64]. Future work will focus on evaluating the effect of different depths of sequencing combined with de novo assembly parameters (e.g., *k*-mer length) to determine their effect on discovery of contaminant-affected transcripts.

Comparative analysis of shared hepatic functions across mackerel, sardine, and turbot demonstrated here also contributes to transcriptomic analysis across species that can be expanded to include other fish species and marine systems. The assembly of de novo transcriptomes bypasses the challenges of finding an appropriate reference genome for non-model fish while also expanding our knowledge of phylogenetic relationships [65,66,67]. Even with advances in transcriptome assembly of non-model organisms, functional annotation relies in part on the detailed research of model organisms. This study however, facilitates a “humanized” interpretation of the effect of contaminants on fish liver function, which may give insight into downstream concerns on human health [55]. Similarities in conserved functions and phylogenetic relationships can be combined to provide further insight into suitable biomarker selection across species and habitats [68,69].

## Supplementary Materials

The following are available online at http://www.mdpi.com/2073-4425/9/11/521/s1, Table S1: Mackerel (*Scomber japonicas*) Trinity annotation for contigs with at least one annotation hit, Table S2: Sardine (*Sardinops sagax*) Trinity annotation for contigs with at least one annotation hit, Table S3: Turbot (*Pleuronichthys verticalis*) Trinity annotation for contigs with at least one annotation hit, Table S4: Quantitative characterization of Trinity de novo assemblies of mackerel (*Scomber japonicas*), sardine (*Sardinops sagax*), and turbot (*Pleuronichthys verticalis*), Table S5: Benchmarking Universal Single-Copy Orthologs (BUSCO) analysis with the Actinopterygii_odb9 [48] dataset with zebrafish as reference for the completeness of assembled transcriptomes for mackerel (*Scomber japonicas*), sardine (*Sardinops sagax*), and turbot (*Pleuronichthys verticalis*), Table S6: List of blastp hits and their overlap from de novo hepatic assembled transcriptomes of mackerel (*Scomber japonicas*), sardine (*Sardinops sagax*), and turbot (*Pleuronichthys verticalis*), Table S7: List of gene ontology biological process identifiers and their overlap from the blastp hits from de novo hepatic assembled transcriptomes of mackerel (*Scomber japonicas*), sardine (*Sardinops sagax*), and turbot (*Pleuronichthys verticalis*) using a zebrafish gene ontology annotation background, Table S8: List of the top 350 significant gene ontology biological process identifiers and p-values from the blastp hits from de novo hepatic assembled transcriptomes of mackerel (*Scomber japonicas*), sardine (*Sardinops sagax*), and turbot (*Pleuronichthys verticalis*) using a zebrafish gene ontology annotation background, Table S9: List of gene ontology biological process identifiers and their overlap from the blastp hits from de novo hepatic assembled transcriptomes of mackerel (*Scomber japonicas*), sardine (*Sardinops sagax*), and turbot (*Pleuronichthys verticalis*) using a human gene ontology annotation background, Table S10: List of the top 350 significant gene ontology biological process identifiers and p-values from the blastp hits from de novo hepatic assembled transcriptomes of mackerel (*Scomber japonicas*), sardine (*Sardinops sagax*), and turbot (*Pleuronichthys verticalis*) using a human gene ontology annotation background, Table S11: List of the gene ontology biological process identifiers (max. 350 terms) and p-values that were unique to each fish from the blastp hits from de novo hepatic assembled transcriptomes of mackerel (*Scomber japonicas*), sardine (*Sardinops sagax*), and turbot (*Pleuronichthys verticalis*) using a human gene ontology annotation background.
